# The use of autologous skeletal muscle progenitor cells for adjunctive treatment of presumptive urethral sphincter mechanism incompetence in female dogs

**DOI:** 10.1111/jvim.16505

**Published:** 2022-08-05

**Authors:** Shelly L. Vaden, Kyle G. Mathews, James Yoo, James Koudy Williams, Tonya Harris, Patty Secoura, James Robertson, Katherine L. Gleason, Hannah Reynolds, Jorge Piedrahita

**Affiliations:** ^1^ Department of Clinical Sciences North Carolina State University Raleigh North Carolina USA; ^2^ Wake Forest Institute for Regenerative Medicine Winston‐Salem North Carolina USA; ^3^ NC State Veterinary Hospital North Carolina State University Raleigh North Carolina USA; ^4^ College of Veterinary Medicine North Carolina State University Raleigh North Carolina USA; ^5^ Department of Molecular Biomedical Sciences North Carolina State University Raleigh North Carolina USA

**Keywords:** canine, regenerative medicine, stem cell, urinary incontinence

## Abstract

**Background:**

Urethral sphincter mechanism incompetence (USMI) is a common problem in female dogs, but some dogs fail to achieve continence with standard treatment. Urethral submucosal injection of autologous skeletal muscle progenitor cells (skMPCs) previously has been shown to restore urethral function in a canine model of USMI.

**Hypothesis/Objective:**

To determine if urethral submucosal injection of skMPC alters continence in dogs with USMI that had previously failed standard medical management. We hypothesized that the injections would lead to improved continence.

**Animals:**

Fifteen client‐owned dogs with USMI that had failed standard medical management.

**Methods:**

Dogs were prospectively enrolled into a single‐armed clinical trial. Once enrolled, a triceps muscle of each dog was biopsied; the tissue specimens were digested, cultured, and expanded to 100 million cells before injection into the urethral submucosa using a surgical approach. Continence was assessed at baseline and 3, 6, 12, and 24 months post‐injection using continence scores and urethral pressure profilometry.

**Results:**

Median continence scores increased significantly from baseline at 3, 6, 12, and 24 months. Increases were seen in 14 of 15 dogs with 7, 6 or 1 dog achieving scores of 5, 4 or 3, respectively. Additional medication was required to achieve continence in all but 2 dogs.

**Conclusions and Clinical Importance:**

Urethral submucosal injection of skMPC can be used adjunctively to improve continence in dogs with difficult to manage USMI. The procedure is labor intensive but well tolerated; most dogs will require continued medication to remain continent.

**Abbreviations**
ANOVA: analysis of variance
s1DES: diethylstilbesterol
DMEM: Dulbecco's Modified Eagle Medium
MUCP: maximal urethral closure pressure
PDS: polydioxanone suture
PPA: phenylpropanolamine
skMPC: skeletal muscle progenitor cells
SUI: stress urinary incontinence
UCP: urethral closure pressure
UPP: urethral pressure profilometry
USMI: urethral sphincter mechanism incompetence

## 1 INTRODUCTION

It is estimated 1 of 5 female dogs will develop urinary incontinence at some point in their lifetime; the incidence is highest in large breed dogs.^1^, ^2^ Urethral sphincter mechanism incompetence (USMI), the most common cause of incontinence in female dogs, is characterized by the unconscious leakage of urine during rest or recumbency and caused by insufficient urethral closure pressures (UCP). Breed, ovariohysterectomy, short urethral length, pelvic bladder, obesity, urinary tract infection, and polyuria are factors that can impact the occurrence or severity of USMI.^1^, ^3^ Historical treatment of USMI has been to augment UCP using alpha‐adrenergic agonists, estrogens, or gonadotropin‐releasing hormone analogues; injection of urethral bulking agents; or, surgery, including an artificial urethral sphincter, urethropexy or colposuspension.^4^, ^5^, ^6^, ^7^, ^8^, ^9^, ^10^, ^11^, ^12^, ^13^ Some dogs fail to achieve continence regardless of the method used. Failure to manage urinary incontinence may lead to dogs being relegated to outdoor living, surrendered to animal shelters, or brought to veterinarians for euthanasia.

Stress urinary incontinence (SUI) is common in women and often the cause of urinary sphincter deficiencies caused by childbirth and aging.^14^, ^15^ Although not the same disorder as USMI in dogs, and dogs and humans have different anatomy, SUI presents clinically as urine leakage during times of increased abdominal pressure (e.g., coughing, sneezing, lifting). Urethral bulking and surgeries are often unsatisfactory and stem cells for regenerative repair of the urethral sphincter have been a focus of recent research into improving treatment of women with SUI and have been shown to be moderately effective for long‐term benefit.^16^, ^17^, ^18^ The use of autologous skeletal muscle progenitor cells (skMPCs) in a canine model of induced USMI has been reported previously.^19^ This study explored the possibility of using autologous skMPC as an injectable, cell‐based treatment in a canine model of USMI, induced by microsurgical removal of the sphincter. Dogs receiving skMPC injections had maximal urethral closure pressures (MUCP) approximately 80% of normal, whereas the pressures in control dogs with surgically‐damaged sphincters but without skMPC injection remained at 20% of normal values. In addition, analysis indicated that the implanted cells survived and formed tissue, including innervated muscle fibers, within the injected region of the sphincter. These results indicated that skMPC can restore otherwise irreversibly damaged urinary sphincter function.

We elected to explore the use of submucosal urethral injection of autologous skMPC in dogs with naturally‐occurring USMI as an alternative treatment for dogs that had failed to respond to standard medical management. Our hypothesis was that urethral submucosal injection of skMPC would result in improved continence in dogs with USMI. Our primary objective was to determine if injection of autologous skMPC into the urethral submucosa as an adjunctive treatment would result in effective long‐term control of urinary incontinence in dogs with USMI that had previously failed standard medical management.

## 2 MATERIAL AND METHODS

### 2.1 Animals

Spayed female dogs that had urinary incontinence at rest and been diagnosed with USMI but failed standard medical management were recruited for this prospective, single‐armed clinical trial. The diagnosis of USMI was confirmed by verifying clinical signs that were consistent with USMI (ie, incontinence at rest and not while walking), failure to document another cause of incontinence through standard clinical evaluation (complete history and physical examination, serum biochemistry panel, CBC, urinalysis, urine culture, abdominal ultrasound examination and vaginocystourethroscopy). Failed medical management was defined as either continued incontinence despite drug administration (i.e., phenylpropanolamine [PPA] or an estrogen (e.g., estriol, diethylstilbestrol [DES]), administered alone or in combination at doses within the recommended ranges published in companion animal formularies) or the presence of unacceptable adverse effects of the medication (e.g., anxious behavior, aggression).^4^, ^5^, ^6^ Dogs were excluded if they had a congenital anatomic cause for their incontinence (e.g., ectopic ureter, congenital vaginal abnormality), a pattern of incontinence that was not compatible with a primary diagnosis of USMI (i.e., if the leakage of urine occurred primarily while walking or after voiding), or unresolved urinary tract infection. The study was approved by the Institutional Animal Care and Use committee of North Carolina State University College of Veterinary Medicine; all participating owners gave informed consent.

### 2.2 Assessment of continence

Continence was assessed at baseline and 3, 6, 12, and 24 months after skMPC injection using a urinary incontinence questionnaire (Figure S1) and urethral pressure profilometry (UPP). Based on the answers the owners provided to the urinary incontinence questionnaire, dogs were assigned a continence score of 1 to 5, with 1 being a dog that is never continent and 5 being a dog that is always continent. Answers to the questionnaire also were used to determine if dogs were experiencing any outward signs that might suggest an adverse reaction to the skMPC injection and to record concurrent medications used to improve continence.

Urethral pressure profilometry was used to measure MUCP as previously described.^20^, ^21^ For the UPP procedure, anesthesia was induced in each dog by administration of propofol (4‐6 mg/kg IV). Immediately afterward, dogs were intubated, connected to a semi‐closed circle system with sevoflurane as the inhalant, and mechanically ventilated. A multi‐gas analyzer (Gas Module SE, Mindray, Mahway, New Jersey) was connected to the endotracheal tube to measure the end‐tidal sevoflurane concentration. The UPP was initiated after at least 30 min had elapsed since injection of propofol and 10 min had elapsed since decreasing the end‐tidal sevoflurane concentration to 2.0%.

During the intervening period between anesthesia induction and UPP initiation, the rectum was manually evacuated and an abdominal sensor catheter (TDOC‐7FA, T‐DOC Co, Wilmington, Delaware) was placed through the anus and into the colon to a point approximately 15 cm from the anus. The vulva and surrounding area were clipped and prepared using betadine solution and saline. Using sterile technique, a dual sensor catheter (TDOC‐7FD, T‐DOC Co, Wilmington, Delaware) was passed through the urethra to the level of the bladder. The system and catheters were prepared following manufacturer instructions that accompanied the urodynamic measurement system (Goby KT, Laborie Medical Technologies, Mississauga, Ontario). The bladder was infused with warmed saline until urine leaked from the patient or, if leakage did not occur, until a volume of 10 mL/kg was achieved. The catheter was clamped to the mechanical puller arm and the puller speed was set to 1 mm/s. The tracing was observed as the recorded urethral pressure increased and then decreased to baseline. The pull was stopped. The procedure was repeated 3 times. Maximal and baseline urethral pressures were recorded. The baseline urethral pressure was subtracted from the maximal urethral pressure to derive the MUCP.

### 2.3 Isolation of skMPCs

After baseline UPP, a triceps muscle biopsy was obtained for harvesting of the skMPC. The plane of anesthesia was increased after UPP to allow for muscle biopsy; protocols varied, as determined by the attending anesthesiologists. Each patient was placed in lateral recumbency, and aseptic preparation of the triceps biopsy site was performed. A 5 cm incision was made through the skin and SC fat using a #10 scalpel blade. Gelpi retractors were placed, exposing the fascia of the lateral head of the triceps muscle. An elliptical incision was made in the triceps using the scalpel and an approximately 1 cm^3^ sample of triceps muscle was removed using Metzenbaum scissors. The biopsy sample was placed in a sterile moist gauze within a sterile specimen cup and submitted for skMPC isolation. The triceps muscle was closed with 2‐3 cruciate sutures using 3‐0 to 4‐0 polydioxanone suture (PDS). The SC tissue was closed with a simple continuous pattern using 3‐0 to 4‐0 PDS. The subcuticular layer was closed in a continuous horizontal mattress pattern with 4‐0 PDS or Monocryl (Monocryl, Ethicon, Cornelia, Georgia). The skin incision was opposed with tissue adhesive.

Biopsy specimens were minced using scalpels and connective tissue was removed. Minced muscle tissue was digested in 0.2% type I collagenase. After digestion, tissue was plated on Matrigel‐coated plates to allow for the expansion of skMPC. As cells expanded, they were passaged 2 to 3 times at 50% to 70% confluence. Cell viability was assessed by visual inspection before harvest for injection; cells were assessed to be viable if they were adherent to the surface of the tissue culture plate, were compact and round to oblong and refracted light when viewed by phase contrast microscopy.^22^ Once cell numbers reached 100 million, cells were harvested and resuspended 0.6 mL of phenol‐free Dulbecco's Modified Eagle Medium (DMEM) in preparation for injection.

### 2.4 Injection of skMPCs

Once adequate cell numbers were reached, the patient was anesthetized using an anesthetic protocol that was determined by the attending anesthesiologists. Each patient was placed in dorsal recumbency, and the surgical site was aseptically prepared. A 10 to 15 cm ventral midline incision was made using a #10 scalpel blade starting in the caudal abdomen and extending to the pubis. The SC tissues were bluntly dissected, and hemostasis was achieved using electrocautery. A 10 cm incision was made in the caudal *linea alba*. The bladder was exteriorized, and a single stay suture was placed in the apex of the bladder. The skin, SC tissues, and linea were retracted using Weitlaner or Gelpi retractors just cranial to the pubis. The proximal urethra was located, and fat was cleared from the ventral and lateral aspects of the urethra using blunt dissection with right‐angle forceps and cautery.

In 3 separate syringes, approximately 0.2 mL of cell suspension was mixed with approximately 0.13 mL of the dog's own plasma immediately before injection. The plasma was added so as to form autologous platelet gel that could provide a nurturing scaffolding for the stem cells.^23^ Each aliquot was injected via a new 25‐gauge needle into the urethral muscularis evenly divided among 3 evenly spaced sites circumferentially around the proximal urethra. One milliliter of material (0.6 mL of cell suspension and 0.4 mL plasma) was injected. Cystourethroscopy was used to visualize the internal lumen of the urethra and verify that the needle did not penetrate through the urethral wall into the lumen. The 3 injection sites were spaced approximately 120° apart, with the first injection on the ventral aspect of the urethra. A bleb of suspension could be visualized within the wall at each site both externally and internally (via the endoscope). The stay suture was removed from the apex of the bladder.

The linea was closed with 0 to 2‐0 PDS in a simple continuous pattern. The linea and SC tissues were blocked with 2 mg/kg of 0.25% bupivacaine. The SC tissue was closed using a simple continuous pattern with 3‐0 PDS. The subcuticular layer was closed in a continuous horizontal mattress pattern with 3‐0 to 4‐0 PDS or Monocryl (Ethicon, Inc., J&J Surgical Technologies, Bridgewater, New Jersey, USA). The skin was opposed using staples or tissue adhesive.

After recovery from general anesthesia, dogs were hospitalized overnight and discharged the next day. If there were no contraindications, carprofen (4.4 mg/kg IV, once) was administered at recovery and then continued PO (2.2 mg/kg q24h) for 3 to 5 days starting 24 h after the IV dose. Antibiotics were administered only if previously prescribed for the treatment of urinary tract infection. Between anesthetic recovery and discharge, the incision was evaluated, and pain scored q4‐6h. A cold compress was applied to the incision q6h. Narcotic analgesia was provided as needed q4‐6h if the pain score was ≥2 out of 4.

Medications used to maintain continence were maintained in the first 3 months post‐injection to allow for tissue regeneration and remodeling and were discontinued 3 months post‐injection. After that time point, medications were administered to improve continence as needed as determined by the attending clinician.

### 2.5 Statistical analysis

An omnibus test was conducted in the form of analysis of variance (ANOVA) on a mixed effects model with time as a categorical variable as the only fixed effect, patient as the only random effect, and the response as the rank of the score within the patient. Where significance was found with the omnibus test, it was followed with paired Wilcoxon tests comparing each time‐point to baseline. Bonferroni adjustment rendered a significance cutoff of .0083 to maintain a 5% false‐positive rate.

## 3 RESULTS

### 3.1 Animals

Between November 2013 and August 2017, 17 spayed female dogs were identified for the study (Table S1). At the time of enrollment, the median age was 4.25 years (range, 1.16‐10.25). There were 6 Doberman pinscher, 2 mixed breed dogs, and 1 dog of each of the following breeds: standard poodle, giant schnauzer, collie, border collie, American Staffordshire terrier, soft‐coated wheaten terrier, Australian shepherd, Labrador retriever, and Puli.

Two dogs were excluded from final data presentation and analysis. In the first dog, an extreme weather event disrupted normal procedures and it was determined by visual inspection of remaining cells immediately after injection that most cells were not viable at the time of injection. The second dog died before the 3‐month re‐evaluation from acute spinal cord injury, unrelated to the study.

For 15 dogs, the owners reported a median and approximate duration of urinary incontinence of 3 years before enrollment (range, 0.5‐9 years); urinary incontinence began at an undetermined time before the remaining 2 dogs being adopted as adults. Medications used that failed to control incontinence before enrollment in the study were PPA (15 dogs), either alone or in combination with diethylstilbestrol (6) or estriol (3). In addition to persistent incontinence, 3 dogs had unacceptable adverse effects that required discontinuation of the medication (aggression with diethylstilbestrol, 1; excessive anxiousness with PPA, 1; vomiting with PPA, 1).

### 3.2 Isolation and injection of skMPCs

After biopsy, skMPC were expanded in culture for a median of 18 days (range, 14‐27 days). For 14 dogs (#4‐17), cell passage at the time of injection was tracked: 8 of 14 were injected at passage 2 and 6 of 14 were injected at passage 3. Cells were harvested on the day of injection, and 100 million cells were resuspended in 0.6 mL phenol‐free DMEM in preparation for injection. One dog (#13) was injected with only 40 million cells because of slow cell growth.

The surgical procedure was uneventful in all dogs; there were no apparent complications. During the first few injections, it was discovered that the needle could become occluded if the injections were not done rapidly and that successful injections subjectively required a high degree of force. This finding deterred us from transferring this procedure to a minimally invasive technique.

### 3.3 Assessment of continence

**FIGURE 1:**
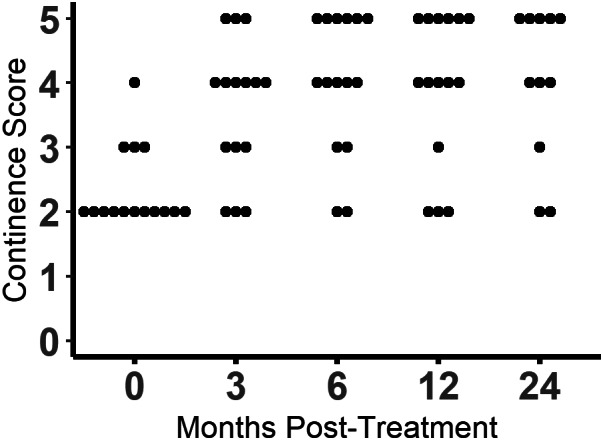
Continence scores at baseline and 3, 6, 12, and 24 months after urethral injection of skeletal muscle progenitor cells. Dogs were given additional medications as needed to improve continence. Each dot represents and individual dog.

**FIGURE 2:**
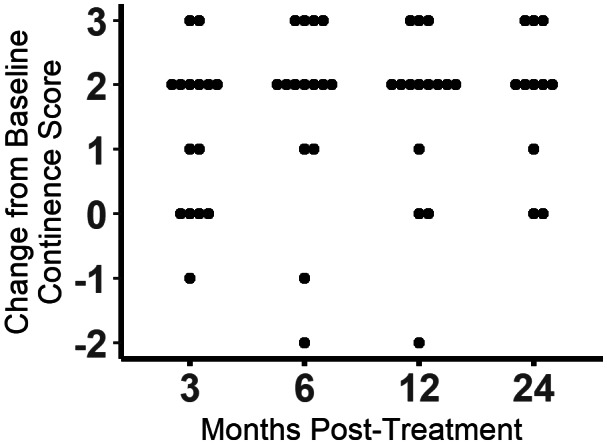
The change in continence scores from baseline at 3, 6, 12, and 24 months after urethral injection of skeletal muscle progenitor cells. Dogs were given additional medications as needed to improve continence. Each dot represents and individual dog.

Continence scores increased at some point at the study in 14 of 15 (93.3%) dogs (Figures 1 and 2; Figure S2). The omnibus test was significant (*P* < .0001). Continence scores were found to be significantly different from baseline at 3 months (*P* = .006), 6 months (*P* = .004), 12 months (*P* = .005), and 24 months (*P* = .008). Of the 14 dogs with increased continence scores, 7 (50%) achieved a score of 5, 6 (42.8%) achieved a score of 4, and 1 only improved to a score of 3. At the 3‐month evaluation, 10 of 15 (66.7%) dogs had improved continence, 4 (26.7%) dogs were unchanged, and 1 dog had worsened incontinence compared to baseline. At the 6‐ and 12‐month evaluations, 13 of 15 (86.7%) dogs had improved continence, 1 dog was unchanged, and 1 dog had worsened incontinence compared to baseline. By 24 months post‐injection, 2 dogs had died from unrelated causes and 2 were lost to follow‐up. Of the remaining 11 dogs, 9 (81.8%) had improved continence, and 2 were unchanged. Throughout the study, 1 dog never experienced improvement and 3 dogs had initial improvement and then returned to their baseline continence scores. Of the dogs that returned to baseline continence, 2 did so at 12 months and 1 did so at 24 months.

Additional medical management was needed to achieve continence in all but 2 dogs. One of these dogs achieved a continence score of 5 and the other achieved a continence score of 4. Of the remaining 13 dogs, 4 were treated with PPA only, 2 were treated with diethylstilbesterol only, 6 were given a combination of phenylpropanolamine and diethylstilbesterol, and 1 was given a combination of phenylpropanolamine and estriol to improve their continence during the observational period that followed the 3‐month post‐injection time point. Of the 13 dogs that needed additional medical management, 9 were given the same medications at the end of the study period that were found to be ineffective in controlling their incontinence before skMPC injection; 5 of these 9 had improvement of their incontinence. Two dogs failed to be continent with a combination of phenylpropanolamine and diethylstilbesterol before skMPC injection but were continent with diethylstilbesterol alone after skMPC injection. The remaining 2 of the 13 dogs requiring medication originally were given phenylpropanolamine but by the end of the study were receiving both phenylpropanolamine and diethylstilbesterol to achieve continence.

For 3 dogs, a malfunction in the UPP procedure occurred at baseline, but measurements were continued at other time points; these dogs were excluded from baseline analyses (Figure S3). One dog was not returned for UPP after 3 months and the data were excluded from analysis. The owners of another dog failed to return the dog for UPP at 3 months, but other time points were available for analysis. For a third dog, the owners did not return for the 3‐ and 6‐month time points but other time points were available for analysis. No difference in UPP scores through time was found using an omnibus ANOVA test (*P* = .41). No significant difference was found at 3 months (*P* = .12), 6 months (*P* = .22), 12 months (*P* = .38), or 24 months (*P* = .58). A relationship was found between continence score and MUCP (*P* = .03) with patients achieving higher MUCP values when achieving higher continence scores. In addition, the highest MUCP measured corresponded to a time when the owner reported the highest continence scores in 10 of 14 dogs and a MUCP higher than baseline corresponded to a time when the owner reported higher continence scores in 11 of 14 dogs.

## 4 DISCUSSION

We found that regenerative medicine, specifically urethral submucosal injection of skMPC, with or without adjuvant medication, can lead to improved continence in dogs with USMI that have failed medical management. In this difficult to treat population, 14 of 15 dogs (93%; 9 dogs at 3 months, 3 at 6 months, and 2 at 12 months) achieved a continence score of 4 (mostly continent) or 5 (always continent) after injection of skMPC, a success rate that is equal or higher than what has been reported using other advanced treatments for USMI in dogs.^7^, ^8^, ^9^, ^10^, ^11^, ^12^, ^13^ In addition, the improvement appears to be enduring given that 11 of 15 dogs (73.3%) still had improved continence, compared with baseline, at 24 months after injection of skMPC. All but 2 of the dogs required concurrent medical treatment to achieve this level of continence, suggesting that in most dogs skMPC injection alone was not curative albeit facilitative. As has been reported in other species, our results indicate that the urethral submucosal injection of skMPC can be used as an adjuvant treatment to achieve continence in dogs.^18^, ^19^, ^24^, ^25^, ^26^

Dogs in our study had already failed standard treatment for USMI but achieved continence rates equal to or exceeding what would have been expected of standard medical treatment in the population of dogs at large with USMI. Standard medical management for USMI is most commonly administration of an alpha‐agonist (e.g., PPA), or an estrogen (e.g., diethylstilbesterol [DES] or estriol) or a combination of the 2 agents.^4^, ^5^, ^6^, ^27^Although a large percentage of patients initially respond well to PPA, prolonged administration results in a reduction in response and some dogs experience intolerable adverse effects from PPA, which can include anxious behavior, systemic hypertension, diarrhea or vomiting.^4^, ^5^, ^28^ Likewise, although estrogens can improve continence in approximately 80% of treated dogs, a large percentage of dogs have some level of persistent incontinence and response may decrease over time.^6^

Dogs in our study achieved continence rates that are similar those previously reported for the advanced therapeutics options that have been developed for dogs with USMI that have failed standard medical treatment. Dogs in our study treated with injection of skMPC did not have any complications and, with additional treatment, the response was enduring over months of observation in most dogs. When submucosal collagen injections were used as a urethral bulking agent, continence was obtained in up to 68% of dogs; administration of additional medication led to continence in 83% of the dogs.^7^ A major drawback of urethral bulking however is the short duration of efficacy, requiring repeat injections and concurrent pharmacologic management.^7^, ^8^ Several surgical approaches to the management of USMI in dogs have been reported. Colposuspension is reported to lead to improved continence in 50% to 65% of dogs when combined with medical management but can lead to urine retention and dysuria.^13^, ^14^ Other surgeries, including urethropexy and transobturator vaginal tape have similar success rates and complications.^12^ More recently, the placement of an artificial urethral sphincter (hydraulic occluder) has become a surgical option for dogs with difficult to treat USMI. A retrospective study reported improved continence scores for all dogs with acquired USMI after placement of an artificial urethral sphincter.^9^ However, 2 dogs in the study had progressive urethral obstruction 5 and 9 months after surgery, ultimately requiring removal of the device.

Although median continence scores increased significantly in the dogs of our study, median MUCP measurements did not. There are several possible explanations for this discrepancy. First, there were many missing data points from the UPP data resulting from equipment malfunctions in 3 dogs and the owners of another 3 dogs failing to return their dogs for repeat UPP measurements. In support of this possibility is the finding that a relationship did exist between continence scores and MUCP, with the highest MUCP measurements corresponding to the time when the owner reported the highest continence scores. Many technical aspects of the UPP procedure can lead to variable results, including catheter size and integrity, volume of fluid in the bladder and anesthesia.^20^, ^21^ We tried to minimize these effects by using a standard protocol, but it is impossible to completely remove the impact of these variables on results. Finally, the owners were not blinded and were subjectively scoring the level of continence in their dogs. This situation potentially added bias to the continence scores that was not present in the evaluation of the UPP results.

Mesenchymal stem cells originating from adult tissues such as bone marrow, adipose tissue, placenta, umbilical cord, and blood have been used commonly to treat and possibly cure SUI associated with sphincter deficiency.^18^, ^29^ Although adult stem cells have a lower differentiation capacity than embryonic cells, they are ideal for regenerative medicine applications because there is a low risk of malignant differentiation and they can be obtained by autologous transfer, thereby eliminating the risk of recipient rejection. In addition, the use of adult stem cells is not associated with ethical controversy.^30^ When adult stem cells are implanted into the external urethral sphincter, it is believed that the damaged musculature is restored by induction of muscle and nerve regeneration via a complex process involving remodeling of the matrix and restoration of the cells.^30^

The use of skMPCs has been shown to provide long‐term improvement in urinary sphincter structure (increased number of muscle cells and decreased fibrosis) as well is improved MUCP in dog, nonhuman primate and rat models of induced urinary sphincter deficiency.^19^, ^24^, ^25^, ^26^ Our study supports these findings and expands the clinical applicability of this regenerative approach in a clinical setting.

Limitations of our study include that it consisted of a relatively small subset of patients (i.e., restricted to dogs that had failed medical management). The potentially achieved continence rates in dogs with USMI from the population at large is unknown, but possibly larger than we observed. Limitations of the procedure include that the methods used require collaboration with a laboratory that is capable of expanding skMPC in culture to allow for the injection of an appropriate number of cells, thereby limiting the availability of the procedure. Ideally, such a procedure would be conducted using minimally invasive procedures. However, the addition of an autologous platelet gel led to rapid clot formation in under a minute. We believe the rapidity at which the injection needle became occluded, and the high level of force required for adequate injection may limit the transference of this procedure to a minimally invasive approach. Because the cells required a patient muscle biopsy and weeks to grow in a laboratory, we believed the risk of not being able to inject the cells via an endoscopic needle and losing the cells altogether was too high and it was prudent to continue with the surgical approach. Whereas previous laboratory studies of skMPC injections identified improved urethral function without additional medications, we did not study the efficacy of skMPC in isolation in this clinical population but rather used adjunctive medications as needed with the goal of maximizing continence. Likewise, a control group or direct comparison group was not included in our study. The criteria used for providing additional medication to dogs after 3 months were not standardized, which added variability to the study.

In summary, our study determined that the urethral submucosal injection of skMPC can be used as adjunctive treatment to achieve improved continence in dogs with difficult to manage USMI, but in most dogs additional medication also is required. Although the procedure is labor intensive, injection of skMPC is well tolerated by the recipient dogs.

## CONFLICT OF INTEREST DECLARATION

Shelly Vaden serves as Associate Editor for the *Journal of Veterinary Internal Medicine*. She was not involved in review of this manuscript. No other authors have a conflict of interest.

## OFF‐LABEL ANTIMICROBIAL DECLARATION

Authors declare no off‐label use of antimicrobials.

## INSTITUTIONAL ANIMAL CARE AND USE COMMITTEE (IACUC) OR OTHER APPROVAL DECLARATION

Approved by the IACUC at North Carolina State University (protocol approved 19‐007‐O).

## HUMAN ETHICS APPROVAL DECLARATION

Authors declare human ethics approval was not needed for this study.

## Supporting information


**Figure S1** Urinary incontinence questionnaire administered to owners at baseline and 3, 6, 12 and 24 months after MPC injection

**Figure S2** Urinary continence scores from individual dogs over time during the observational period. All dogs received supplemental medication to maintain continence except dogs 2 and 14.

**Figure S3** Maximal urethral closure pressures (cmH_2_0) from individual dogs over time during the observational period. All dogs received supplemental medication to maintain continence except dogs 2 and 14.

**Table S1** Description of dogs enrolled in study.
