# Supplementary material for: The use of autologous skeletal muscle progenitor cells for adjunctive treatment of presumptive urethral sphincter mechanism incompetence in female dogs
Source: J Vet Intern Med. 2022 Aug 5;36(5):1686–92. doi: 10.1111/jvim.16505 (PMC9511066; doi:10.1111/jvim.16505)
Supplement: Supplementary file 1 — Figure S1 Urinary incontinence questionnaire administered to owners at baseline and 3, 6, 12 and 24 months after MPC injection [file JVIM-36-1686-s002.pdf]

**Please answer all questions based on your dogs behavior within the past 2 weeks as compared to before implant procedure.**

| | **Comments** |
|---|---|
| Activity level during the day:<br>Increased Decreased Same | |
| Activity level at night:<br>Increased Decreased Same | |
| Pacing, agitation:<br>Increased Decreased Same | |
| Panting:<br>Increased Decreased Same | |
| Irritability, aggression:<br>Increased Decreased Same | |
| Barking, vocalization:<br>Increased Decreased Same | |
| Muscle tremors, shaking:<br>Increased Decreased Same | |
| Lethargy:<br>Increased Decreased Same | |
| Appetite:<br>Increased Decreased Same | |
| Water consumption:<br>Increased Decreased Same | |

Please list any other behavioral issues in your dog:

**Urination behavior checklist**

| | **Never** | **Rarely** | **Usually** | **Always** |
|---|---|---|---|---|
| Pet is conscious of its urination (postures to urinate) | | | | |
| Pet is not aware of urination (dribbles urine while walking, etc.) | | | | |
| Signals to go outside | | | | |
| Urinates indoors in view of owner | | | | |
| Urinates indoors when owner is not present | | | | |
| Urinates when owner stands over or reaches for the dogs | | | | |
| Urinates indoors when excited | | | | |
| Urinates in many different areas of the house | | | | |
| Urinates in the same spot in the house (when awake) | | | | |
| Urinates in sleeping area during sleep (crate, floor, bedding) | | | | |
| Urinates moderate to large amounts indoors | | | | |
| Urinates small amounts indoors | | | | |

List any incident(s) that has occurred in the past 2 weeks:

**Please circle ONE that best describes your dog**

| Score | Description |
|---|---|
| 1 | Dog is never continent. |
| 2 | Moderately incontinent- leaks urine when laying down, sleeping and/or when bladder is full. |
| 3 | Mildly incontinent- leaks only when sleeping, does not leak when awake. |
| 4 | Mostly continent- leaks urine 1-2 times per week. |
| 5 | Dog is always continent. |

Please list all medications and doses your dog is currently getting:

Figure 1. Urinary incontinence questionnaire administered to owners at baseline and 3-, 6-, 12- and 24-months after MPC injection
