# Supplementary figures and images for: The use of autologous skeletal muscle progenitor cells for adjunctive treatment of presumptive urethral sphincter mechanism incompetence in female dogs
Source: J Vet Intern Med. 2022 Aug 5;36(5):1686–92. doi: 10.1111/jvim.16505 (PMC9511066; doi:10.1111/jvim.16505)

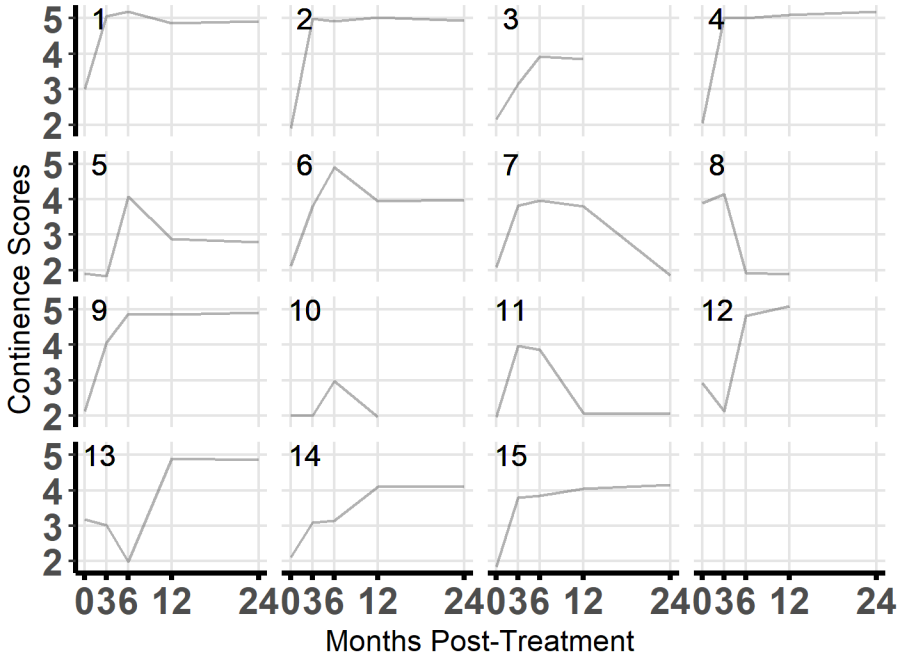

Supplement: Supplementary file 2 — Figure S2 Urinary continence scores from individual dogs over time during the observational period. All dogs received supplemental medication to maintain continence except dogs 2 and 14. [file JVIM-36-1686-s003.pdf]

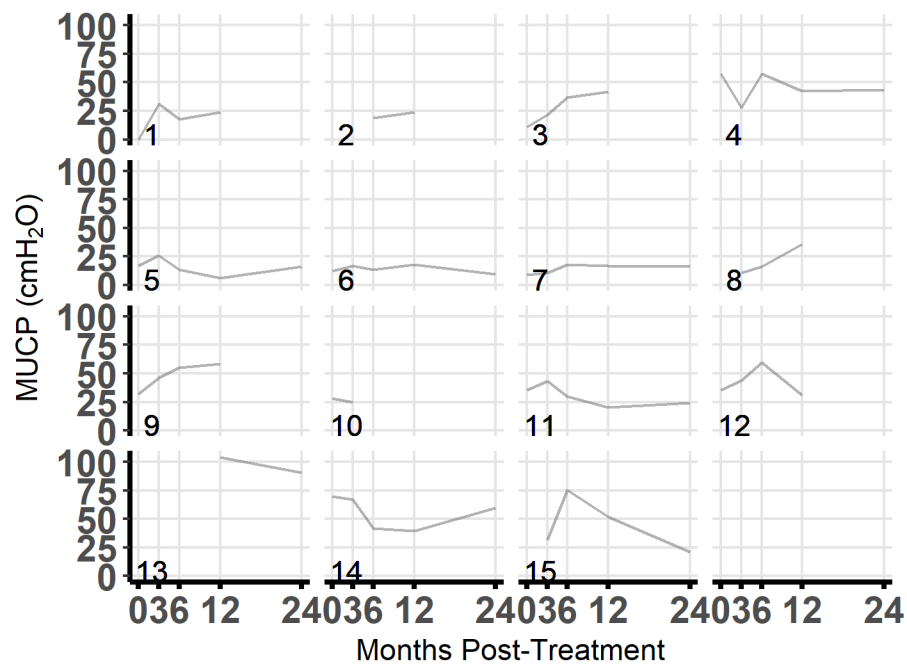

Supplement: Supplementary file 3 — Figure S3 Maximal urethral closure pressures (cmH20) from individual dogs over time during the observational period. All dogs received supplemental medication to maintain continence except dogs 2 and 14. [file JVIM-36-1686-s001.pdf]
