# Supplementary material for: The use of autologous skeletal muscle progenitor cells for adjunctive treatment of presumptive urethral sphincter mechanism incompetence in female dogs
Source: J Vet Intern Med. 2022 Aug 5;36(5):1686–92. doi: 10.1111/jvim.16505 (PMC9511066; doi:10.1111/jvim.16505)
Supplement: Supplementary file 4 — Table S1 Description of dogs enrolled in study. [file JVIM-36-1686-s004.pdf]

Table 1. Description of dogs enrolled in study.

Dog	Breed	Age at enrollment (years)	Approximate duration of incontinence (years)	Medication(s) previously given for urinary incontinence (dose)	Incontinence score at baseline (medication, dose)	Highest continence score (months)	Continence score at last time point (months) (medication dose)
1	mixed breed	2.50	0.67	PPA[a] (1.7 mg/kg q12h)	3 (PPA, 1.7 mg/kg q12h)	5 (3-24)	5 (24) (PPA, 1.7 mg/kg q12h)
2	Doberman pinscher	6.50	5	PPA (1.2 mg/kg q12h-24h), DES[b] (.02 mg/kg q7d)	2 (none)	5 (3-24)	5 (24) (none)
3[c]	American Staffordshire terrier	2.50	2.5	PPA (1.5 mg/kg q8h)	2 (PPA, 1.5 mg/kg q8h)	4 (6-12)	4 (12) (PPA, .5 mg/kg q12h; DES, 1mg q48h)
4	border collie	3.58	3	PPA (1.5 mg/kg q12h), DES (.06 mg/kg q7d)	2 (PPA, 1.5 mg/kg q12h; DES, .06 mg/kg q7d)	5 (3-24)	5 (24) (PPA, 1.5 mg/kg q12h)
5	Doberman pinscher	3.00	2	PPA (1.5 mg/kg q12h)	2 (PPA, 1.5 mg/kg q12h)	4 (6)	3 (24) (PPA, 2.2 mg/kg q12h)
6	Doberman pinscher	6.08	5.3	PPA (1-2 mg/kg q24h)	2 (PPA, 1 mg/kg q24h)	5 (6)	4 (24) (PPA 2 mg/kg q12h)
7	Doberman pinscher	4.25	unknown	PPA (1.0 mg/kg AM and 2.0 mg/kg PM)	2 (PPA, 1 mg/kg AM and 2 mg/kg PM)	4 (3-12)	2 (24) (PPA 2 mg/kg q12h)
8[c]	Doberman pinscher	4.33	4	PPA (3.2 mg/kg q12h), DES (dose unknown), estriol (.06 mg/kg q24h)	4 (PPA, 3.2 mg/kg q12h; estriol (.06 mg/kg q24h)	4 (3)	2 (12) (PPA, 1.7 mg/kg q12h)
9	soft coated wheaten terrier	4.33	4	PPA (.8 mg/kg AM and 1.6 mg/kg PM)	2 (PPA, .8 mg/kg AM and 1.6 mg/kg PM)	5 (12-24)	5 (24) (PPA, 1.7 mg/kg q12h; DES, .07 mg/kg q7d)
10[d]	Australian shepherd	10.25	unknown	PPA (2.1 mg/kg q12h), DES (dose unknown)	2 (none)	3 (6)	2 (12) (PPA, 3.1 mg/kg q12h; DES, .04 mg/kg q7d)
11	Labrador retriever	5.16	3	PPA (2.7 mg/kg q8h), estriol (dose unknown)	2 (PPA, 2.7 mg/kg q8h)	4 (6-12)	2 (24) (PPA, 2.7 mg/kg q8h; DES, .04 mg/kg q7d)

12[d]	Puli	4.00	3.3	PPA (1.3-2.7 mg/kg q12h), estriol (dose unknown)	3 (none)	5 (6-12)	5 (12) (DES .06 mg/kg q7d)
13	giant schnauzer	1.16	0.75	PPA (.7 mg/kg q12h), DES (.03 mg/kg q5days)	3 (DES, .03 mg/kg q7d)	5 (12-24)	5 (24) (PPA, .8 mg/kg q12h; DES, .03 mg/kg q7d)
14	mixed breed	3.04	0.5	PPA (dose unknown), estriol (.03 mg/kg q24h)	2 (estriol, .03 mg/kg q24h)	4 (12-24)	4 (24) (none)
15	standard poodle	3.97	2	PPA (dose unknown), DES (.05 mg/kg q48h)	2 (PPA, 2.2 mg/kg q12h; DES, .04 mg/kg q48h)	4 (3-24)	4 (24) (PPA, 2.2 mg/kg q12h; DES, .04 mg/kg q48h)

a. PPA, phenylpropanolamine, given PO
b. DES, diethylstilbestrol, given PO
c. Died before 24-month evaluation
d. Lost to follow-up
